# Characterization of an Alkaline GH49 Dextranase from Marine Bacterium *Arthrobacter* *oxydans* KQ11 and Its Application in the Preparation of Isomalto-Oligosaccharide

**DOI:** 10.3390/md17080479

**Published:** 2019-08-19

**Authors:** Hongfei Liu, Wei Ren, Mingsheng Ly, Haifeng Li, Shujun Wang

**Affiliations:** 1 Jiangsu Key Laboratory of Marine Bioresources and Environment/Jiangsu Key Laboratory of Marine Biotechnology, Jiangsu Ocean University, Lianyungang 222005, China; 2 Collaborative Innovation Center of Modern Bio-Manufacture, Anhui University, Hefei 230039, China; 3 Co-Innovation Center of Jiangsu Marine Bio-Industry Technology, Jiangsu Ocean University, Lianyungang 222005, China; 4 Medical College, Hangzhou Normal University, Hangzhou 311121, China

**Keywords:** dextranase, marine bacteria, expression, isomalto-oligosaccharide (IMO)

## Abstract

A GH49 dextranase gene DexKQ was cloned from marine bacteria *Arthrobacter oxydans* KQ11. It was recombinantly expressed using an *Escherichia coli* system. Recombinant DexKQ dextranase of 66 kDa exhibited the highest catalytic activity at pH 9.0 and 55 °C. kcat/Km of recombinant DexKQ at the optimum condition reached 3.03 s^−1^ μM^−1^, which was six times that of commercial dextranase (0.5 s^−1^ μM^−1^). DexKQ possessed a *K*m value of 67.99 µM against dextran T70 substrate with 70 kDa molecular weight. Thin-layer chromatography (TLC) analysis showed that main hydrolysis end products were isomalto-oligosaccharide (IMO) including isomaltotetraose, isomaltopantose, and isomaltohexaose. When compared with glucose, IMO could significantly improve growth of *Bifidobacterium longum* and *Lactobacillus rhamnosus* and inhibit growth of *Escherichia coli* and *Staphylococcus aureus*. This is the first report of dextranase from marine bacteria concerning recombinant expression and application in isomalto-oligosaccharide preparation.

## 1. Introduction

Dextranase (EC 3.2.1.11) catalyzes the hydrolysis of dextran (α-1,6-glucosidic glucan). It is classified as endo- and exo-dextranase based on the mode of action. At present, dextranase is mainly applied in industrial sugar processes and is an agent of antidental plaque [1,2]. Many microorganisms, including mold, yeast, and bacteria, can produce dextranase [1]. Compared to its terrestrial counterpart, marine bacteria could produce enzymes with novel properties. Many new polysaccharide hydrolases, including chitinase, chitosanase, alginate lyase, agarose, and carrageenase, have been found in marine microorganisms [3], but there are few reports about marine bacteria producing dextranase. *Arthrobacter oxydans* KQ 11 isolated from China coastal sediment could produce a highly active dextranase, which has been studied in our previous report [4]. Due to the low enzyme yield in native *Arthrobacter oxydans* strains, the application of this dextranase was limited. In this study, a gene coding this native dextranase was cloned and recombinantly expressed using an *Escherichia coli* system. Some characterization and applications of recombinant dextranase were investigated in this study.

## 2. Results

### 2.1. Gene Cloning

In our previous study, a native extracellular dextranase enzyme protein of *Arthrobacter oxydans* KQ11 was purified from its culture supernatant and characterized. Now, gene cloning and recombinant expression of this enzyme were investigated in this study. The protein was digested by trypsin, and the peptide fragment was processed by MALDI-TOF-MS. After analysis of MS results with online Mascot software (www.matrixscience.com), the sequences of some internal peptides of native dextranase are shown in Table 1.

After genomic sequencing of *Arthrobacter oxydans* KQ11 was finished, draft genomic sequence data of 4.4 million bases were analyzed. A total of 4562 ORFs encoding proteins were predicted and annotated. Amino acid sequences encoded by all ORFs were shown one by one in an excel file. All internal peptide sequences shown in Table 1 were found in amino acid sequences coded by *ORF 4194*. None of these internal peptide sequences could be found in any other ORF-coding amino acid sequences. *ORF 4194* was estimated as the coding gene of native dextranase in *Arthrobacter oxydans* KQ11 that we have reported.

### 2.2. Bioinformatic Analysis

**Figure 1:**
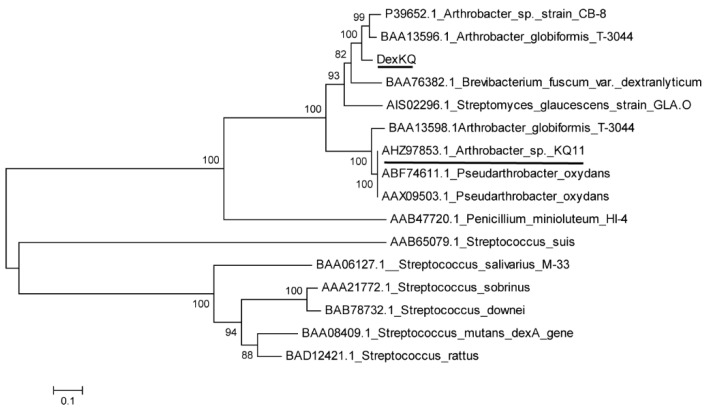
Phylogenetic tree constructed by the neighbor-joining method.

*ORF 4194* of 1923 bp was named DexKQ and registered in Genbank with accession number MK118723. This gene coded a peptide of 640 amino acids with a calculated molecular weight (MW) of 71 kDa. This protein also harbored a predicted signal peptide of 28 amino acid residues. DexKQ was estimated to be an extracellular dextranase coding gene. Most reported dextranases are mainly classified into two glycosyl-hydrolase families of GH 49 and 66 (http://www.cazy.org) [5]. This dextranase encoding by DexKQ was named DexKQ and classified into GH49 according to an online analysis in the CAZy database. Phylogenetic tree results based on the amino acid sequence showed DexKQ had the most similar relationship with two dextranases of P39652.1 and BAA13596.1, which both come from *Arthrobacter* (Figure 1). There were some reports of these two dextranases. Based on N-terminal sequence results of the purified native protein, *BAA13596.1* from *Arthrobacter globiformis* T-3044 was sequence-analyzed and estimated as a coding gene of extracellular native GH49 dextranase [6]. Another GH49 dextranase gene of *BAA13598.1* was also cloned from this strain. This gene was estimated as a silent gene [7]. Recombinant expressions of *BAA13596.1* and *BAA13598.1* in *E. coli* were not studied further. A GH 15 glucodextranase gene in this strain was the main study target of research in Ref. [6]. For dextranase of P39652.1 from *Arthrobacter* sp. CB-8, it was recombinantly expressed in *E. coli.* Recombinant dextranase was stable under neutral or slightly acidic conditions, and it could degrade water-insoluble glucan better than dextranase from *Penicillum* [8]. In our unreported study we cloned a hypothetical dextranase gene from *Arthrobacter oxydans* KQ11, and this gene sequence was registered in Genbank with accession number AHZ97853.1. But it was interesting that the recombinant expressed protein of AHZ97853.1 using the *E. coli* system did not exhibit activity against any dextran substrate. In the phylogenetic tree there was also a hypothetical dextranase gene *AODex* from *Arthrobacter oxydans,* which has the Genbank number AAX09503.1/AY769086 and ABF74611.1 [8,9]. There is high phylogenetic similarity among these four genes—*BAA13598.1*, *AAX09503.1*, *ABF74611.1* and *AHZ97853.1* (Figure 1). We concluded that they all looked like silent or pseudo genes with unknown roles in organisms. In the phylogenetic tree we found that the new-found dextranase gene DexKQ had low sequence similarity with these three dextranase genes (underlined in Figure 1).

**Figure 2:**
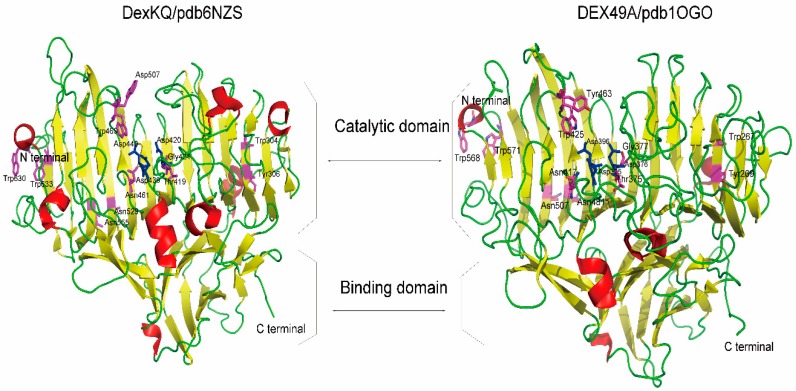
Three-dimensional crystal structure model of DexKQ and DEX49A.

The crystal structure of DexKQ (PDB ID 6NZS) was supplied in our newest published paper [10]. The crystal structure of DexKQ (PDB ID 6NZS) had many similarities with a structure (PDB ID 1OGO) that belongs to GH 49 dextranase DEX49A from *Penicillium minioluteum* [11]. The two structures are shown and compared in Figure 2.

They all consisted of two domains of many β-sheets and several α-helixes. According to a previous report, a total of 14 conserved residues in DEX49A and corresponding residues in DexKQ were found and are listed in Table 2 [11]. Three conserved aspartic acid triads of GH 49 dextranase in DEX49A from *Penicillium minioluteum* were Asp376, 395, and 396. The corresponding key aspartic acid triads in DexKQ were Asp420, 439, 440, which are the top three highlighted in Table 2. In their structures, these amino acid sites in the interior of the β-sandwich are highlighted in purplish-red, and the three GH 49 conserved aspartic acid triads are highlighted by blue. In Figure 2, more surface loops could be found in the structure of DEX49A than in that of DexKQ. Further studies are needed to clarify if these loops are related to better thermal stability of DEX49A.

**Figure 3:**
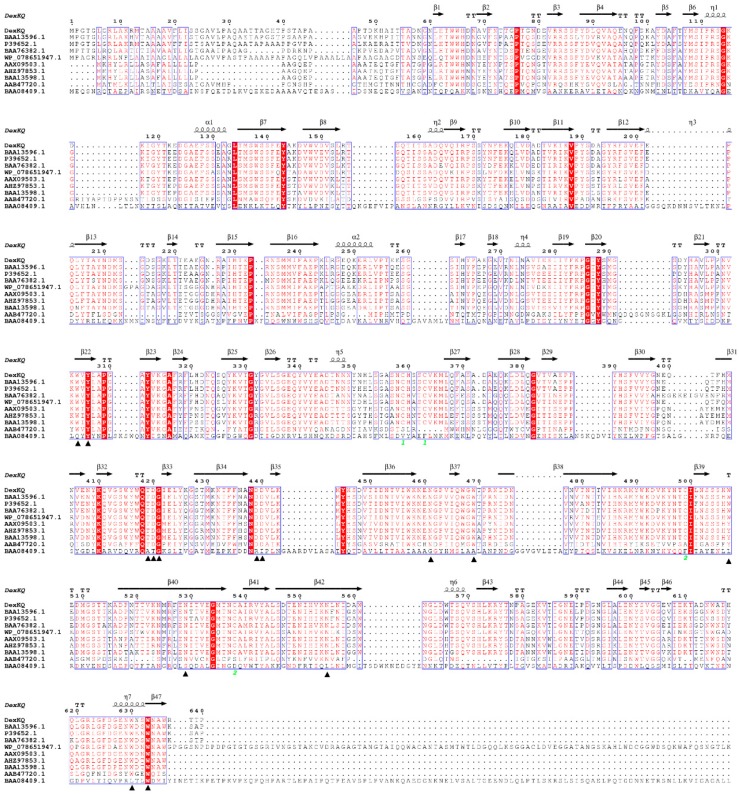
Amino acid sequence alignment of dextranases and secondary structure analysis of DexKQ. DexKQ: GH49 *Arthrobacter oxydans* KQ11, this study; BAA13598.1: GH49 *Arthrobacter globiformis* T-3044; BAA13596.1: GH49 *Arthrobacter globiformis* T-3044; AHZ97853.1: GH49 *Arthrobacter oxydans* KQ11; P39652.1: GH49 *Arthrobacter* sp. CB-8; AAX09503.1: GH49 *Arthrobacter oxydans*; BAA76382.1: GH27 *Brevibacterium fuscum* var. dextranlyticum; AAB47720: GH49 (DEX49A) *Penicillium minioluteum*; BAA08409.1: GH66 *Streptococcus mutans*; WP078651947: GH49 *Streptomyces globisporus*; Amino acid residues that are conserved in all sequences are all labeled in red. Fourteen conserved amino acids including key aspartic acid catalytic triad dextranase of DexKQ (D440, 439, 420) are marked with black triangles.

A two-domain structure is common in dextranase of GH49 but rare in members of other families (GH66, GH27). For example, a GH66 dextranase of *Streptococcus mutants* has a structure with five domains [12]. Isomaltose-dextranase AgIMD (GH27) consisted of the following three domains: A, C, and D [13]. Crystal structure characteristics of DexKQ also proved it to be a typical member of GH49. In order to compare DexKQ with reported dextranase at the primary structure, multiple alignments of dextranase amino acid sequences registered in Genbank were constructed using the software package Clustalx1.83 and accomplished with the ESPript3.0 network station (Figure 3) [14]. A secondary structure analysis of DexKQ based on the three-dimensional structure was accomplished with the ESPript 3.0 network station and is shown in Figure 3. In alignment with reference dextranase sequences from fungi, bacteria, and streptomyces, several conserved regions were found and labeled in red (Figure 3). Fourteen completely conserved residues were found and marked with black triangles. This kind of secondary structure rich in β-sheets was a feature of GH49 dextranase [10,11].

In Figure 3 we can also find that DexKQ had the highest similarity with BAA13596.1 and P39652.1, two GH49 dextranases from *Arthrobacter* strains. On the other hand, DexKQ had low sequence similarities with AAB47720(DEX49A), a GH49 dextranase from *Penicillium minioluteum,* and BAA08409.1, a GH66 dextranase from *Streptococcus mutan*s. We also found the above-mentioned three hypothetical silent genes, *AHZ97853.1*, *AHZ97853.1*, and *BAA13598.1*, had high sequence similarity.

### 2.3. Recombinant Enzyme Expression and Property Characterization

**Figure 4:**
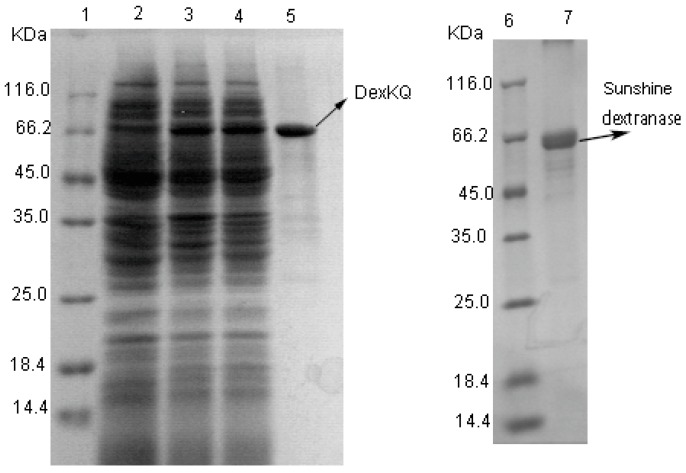
Sodium dodecyl sulfate-polyacrylamide gel electrophoresis (SDS-PAGE) analysis of DexKQ recombinant expression using *E. coli* system. Lane 1: Protein marker; lane 2: Cell lyase without isopropyl β-D-Thiogalactoside (IPTG) induction; lane 3: Cell lyase with IPTG induction; lane 4: Supernatant of cell lyase with IPTG induction; lane 5: Purified DexKQ recombinant enzyme using Ni-IDA resin; lane 6: Protein marker; and lane 7: Dextranase from Sunshine.

DexKQ dextranase was successfully recombinantly expressed and purified using the *E. coli* system. SDS-PAGE results are shown in Figure 4.

**Figure 5:**
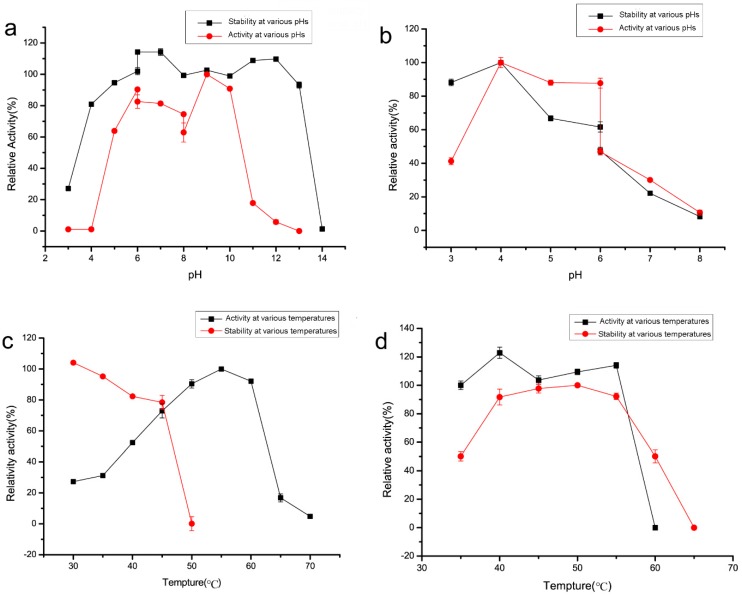
Comparison of temperature and pH adaption of DexKQ and Sunshine commercial dextranase. (**a**) pH adaption of DexKQ; (**b**) pH adaption of Sunshine commercial dextranase; (**c**) NaAc-HAc buffer (pH 3.0–6.0); phosphate salt buffer (pH 6.0–8.0), glycine-NaOH buffer (pH 8.0–13.0); (**d**) temperature adaption of DexKQ, temperature adaption of Sunshine commercial dextranase.

The MW of DexKQ recombinant protein was estimated to be 66 kDa because the band of DexKQ appeared at the same position of a 66 kDa protein in the standard. As a control, commercial dextranase produced by Sunshine Co. Ltd. (Beijing, China) has an estimated MW of 65 kDa. In order to estimate the potentiality of recombinant DexKQ in application, this enzyme was characterized on its catalytic activity, pH, and temperature adaption. Commercial dextranase was also tested as control. Recombinant DexKQ dextranase exhibited the highest catalytic activity at pH 9.0 and showed 80%–100% of its highest activity at pH 7.0–10.0 (Figure 5a). It also had high alkaline tolerance. After incubating at 50 mM in a glycine-NaOH buffer of pH 9.0–13.0 for 2 h at 30 °C, it could maintain at least 90% of initial activity (Figure 5a). Meanwhile, commercial dextranase lost activity quickly when incubated at a pH over 6.0 (Figure 5b). The optimum catalytic temperature of DexKQ dextranase was 55 °C. Its thermal stability was medium. After 120 min of incubation at 45 °C, it also could maintain 76.6% of initial activity (Figure 5c). When the incubation temperature was over 50 °C, it lost activity quickly. Comparatively, commercial dextranase exhibited the highest activity at 40 °C and had better thermal stability than DexKQ (Figure 5d). It maintained almost 90% of its initial activity after incubation at a temperature of 45–55 °C for 120 min. There were some different properties between recombinant DexKQ and wild dextranase [4]. Wild dextranase remained at more than 60% activity at 60 °C for 1 h. Recombinant enzymes, after 2 h of incubation at 45 °C, could maintain 76.6% of initial activity (Figure 5c). When the incubation temperature was over 50 °C it lost activity quickly. Data in our previous paper also revealed the wild enzyme remained at more than 80% residual activity after 2 h of incubation at 30 °C and pH of 7–10. But when the pH was improved to a pH of 11.0, it became unstable. For the recombinant enzyme after incubation in 50 mM glycine-NaOH buffer of pH 9.0–13.0 for 2 h at 30 °C, it could maintain at least 90% of its initial activity (Figure 5a).

**Figure 6:**
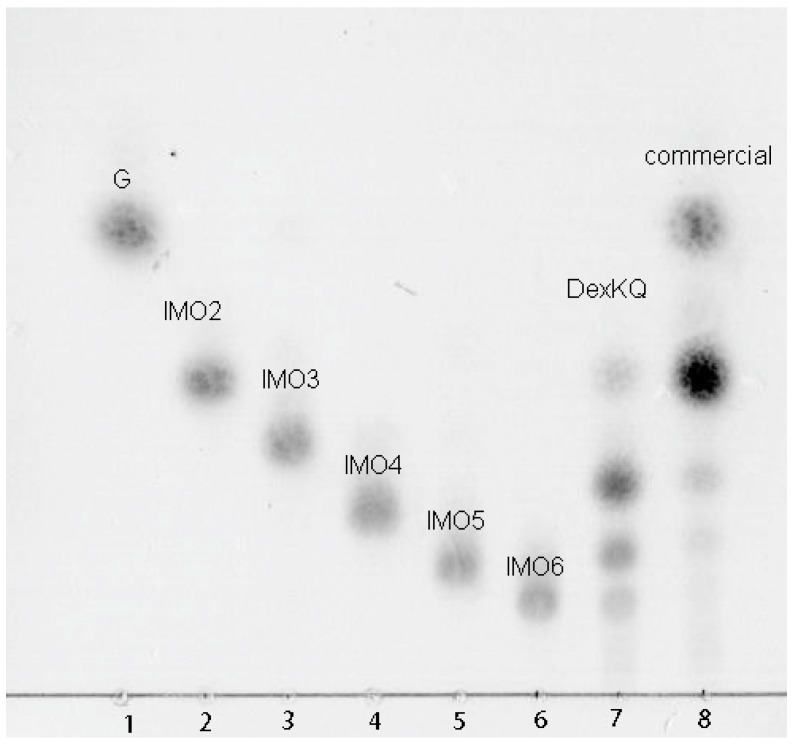
Thin-layer chromatography (TLC) analysis of hydrolysis end products. Lane 1: glucose; Lane 2–6: isomaltose, isomaltotriose, isomaltotetraose, isomaltopentaose, and isomaltohexaose; Lane 7: hydrolysis product of DexKQ; and Lane 8: hydrolysis product of commercial dextranase.

DexKQ was classified as endodextranase because its main hydrolysis end products were composed of 4–6 polymerization-degree isomaltose oligosaccharides (IMO4-IMO6) (Figure 6). Wild enzymes cannot digest nondextran substrates including pullulan, which is a starch without dextran. The main end-products of the wild enzyme against dextran were also isomaltotetraose, isomaltopentaose, and isomaltohexaose [4]. Its properties are the same as those of the recombinant enzyme. The main hydrolytic end products of Sunshine commercial dextranase were glucose and isomaltose (IMO2). DexKQ is superior to the Sunshine enzyme in preparation of isomalto-oligosaccharide with a higher polymerization degree (4–6) when using dextran as substrate. Product distribution without isomaltotriose indicated that the enzymatic hydrolysis mode of DexKQ was different than the other dextranases. This unique hydrolysis mode of DexKQ is worth studying in the future.

Some catalytic properties of recombinant DexKQ and other reported dextranases are shown in Table 3.

**Figure 7:**
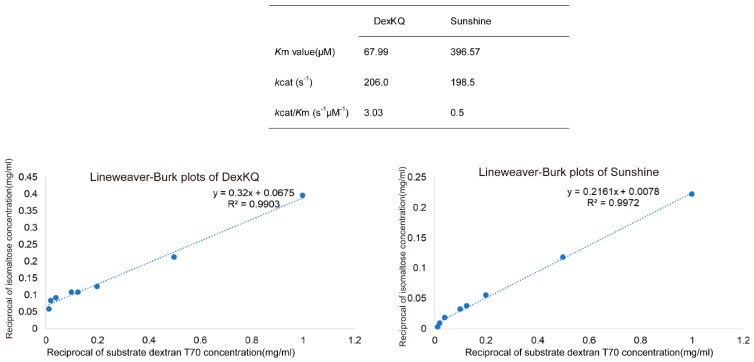
Kinetic parameters of DexKQ dextranase and Sunshine commercial dextranase toward dextran T70. Enzyme activity was determined at optimum conditions of 50 °C and pH 9.0 (for DexKQ) and pH 5.0 (for Sunshine); kcat was calculated assuming a molecular mass of 71 kDa and 65 kDa for DexKQ dextranase and Sunshine dextranase, respectively.

All known dextranases could be classified into four enzyme families: GH15, 27, 49, and 66. Bacterial dextranases could be found in any one of the four GH families [6,7,8,9,15,16,17,18,19,20,21]. All known dextranases from fungi were classified into GH49 [22,23,24,25,26,27]. Fungal dextranases usually were acidic enzymes with optimal pH of 4.5–6.0 and were not stable under high pH conditions. However, there were two exceptions: one was a native dextranase from *Talaromyces pinophilus,* which was stable under pH 3.0–10.0 [23], and the second one was native dextranase from *Fusarium* sp., which was completely stable at a pH ranging from 4.5 to 11.8 under 4 °C [29]. For temperature adaption, dextranase from fungi usually has good activity and stability at 50–60 °C [24,25,26,27]. All known bacteria dextranases were found from Gram-positive bacteria, such as *Bacillus*, *Streptococcus*, *Arthrobacter,* and *Streptomyces* (Table 3). And bacterial dextranases mainly come from GH49 and 66. It was noteworthy that several GH49 endo-dextranases with alkaline tolerance were found in bacteria of *Arthrobacter* and *Streptomyces*, which all belong to actinobacteria in microbial taxonomy. Dextranases of GH66 usually had optimum activity at acidic or neutral conditions [15,16,17,18,19]. Isomaltose exo-dextranase of GH27 and glucodextranase of GH15 had similar properties with GH66 dextranase. Bacterial dextranase from GH49 usually had good pH adaption and showed high activity in broad pH conditions [2,6,30,31]. DexKQ dextranase had the highest alkaline tolerance among the known dextranases in Table 3. After incubation at pH 9.0–13.0 for 2 h at 30 °C, it could maintain at least 90% of initial activity (Figure 5a). This property made it an ideal choice in the alkaline dextran process, other than fungal-derived dextranase like Sunshine dextranase. The lower Km value (67.99 μM vs. 396.57 μM) against the T70 substrate means DexKQ has a higher substrate affinity than the Sunshine commercial enzyme (Figure 7). Additionally, a higher kcat/Km value (3.03 s^−1^ μM^−1^ vs. 0.5 s^−1^ μM^−1^) against the T70 substrate indicated DexKQ had a better dextran hydrolysis capacity than Sunshine dextranase (Figure 7). Lineweaver–Burk plots are provided in Figure 7. However, the catalytic ability of DexKQ was not the highest among dextranases in Table 3. A fungal GH49 family dextranase—dexA, heterologously expressed in two recipient strains of *Penicillium* species—possessed a high specific activity of 1020–1340 U/mg, 1.17–1.18 g/L of Km, and 660–700 s^−1^ of kcat against dextran T70 [27]. And like Sunshine commercial dextranase, dexA is an acidic dextranase with optimal activity at pH 4.5–5.0. Comparatively, DexKQ exhibited 80% of total activity at a wide pH range of 6.0–10.0 (Figure 5). In our published paper, we purified wild dextranase of DexKQ and characterized it [4]. There were some different properties between the recombinant enzyme and wild enzyme. Wild dextranase remained at more than 60% activity at 60 °C for 1 h. For the recombinant enzyme, after 2 h of incubation at 45 °C, it also could maintain 76.6% of its initial activity (Figure 5c). When the incubation temperature was over 50 °C it lost activity quickly. Data also revealed the wild enzyme remained at more than 80% residual activity after 2 h of incubation at 30 °C and at a pH of 7–10. But when the pH was improved to pH 11.0, it became instable. For the recombinant enzyme after incubation in 50 mM glycine-NaOH buffer of pH 9.0–13.0 for 2 h at 30 °C, it could maintain at least 90% of its initial activity (Figure 5a). Wild enzymes cannot digest nondextran substrates that accept dextran. These differences between the wild enzyme and recombinant enzyme need to be studied in the future. It was very interesting that the end products of the recombinant and wild DexKQ dextranase did not contain isomaltose and isomaltotriose, which were found in the end products of every dextranase in Table 3. Thus, it was indicated that the catalytic mechanism of DexKQ was most likely different than all known dextranase.

DexKQ only could hydrolyze continuous α-1,6 glycosidic bonds in the dextran substrate with different MWs. Nondextran substrates, such as pullulan and amylose with single α-1,6 glycosidic bonds, could not be hydrolyzed at all. Other substrates with α-1,6;1,4;1,2 and β-1,4 glycosidic bonds could not be hydrolyzed either (Table 4). As a control, Sunshine commercial dextranase also had strict substrate specificity like DexKQ (Table 4).

### 2.4. Preparation of Isomalto-Oligosaccharide and Application

**Figure 8:**
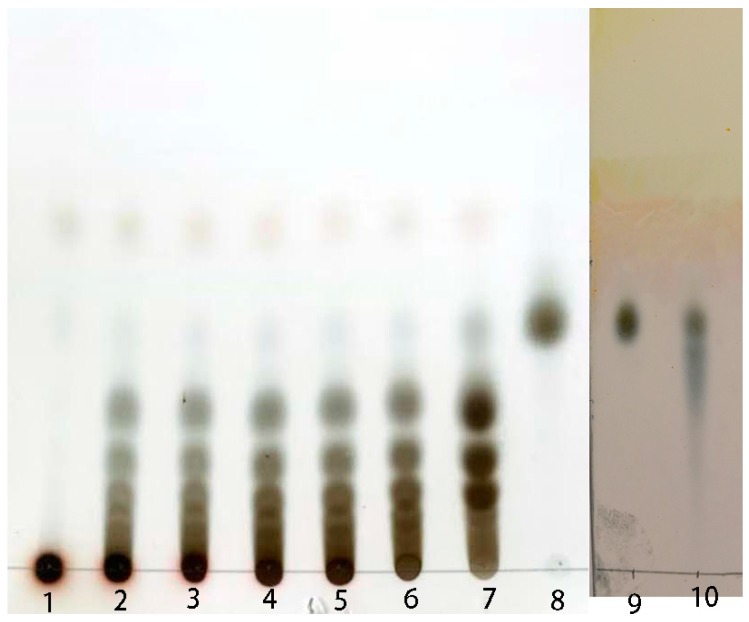
TLC test of samples in preparation of IMO. 1–7 represent samples of 0 min, 0.5, 1, 2, 4, 6, and 12 h; 8 and 9 represent isomaltose standard; 10 represents IMO purchased from market.

During the catalytic course, samples of the reaction mixture were collected at 2–4 h intervals and tested using TLC. After 12 h of incubation, substrates were hydrolyzed completely, and the main products were estimated as IMO3–IMO6 (Figure 8). As a control, IMO purchased from market consisted of IMO2 and a small amount of IMO3. There have been some previous reports about enzymatic hydrolysis dextran into IMO [32,33]. In one report, immobilized GH49 dextranase from *Penicillium lilacinum* was used to digest dextran into IMO at pH 4.5–5.5 and 30–35 °C.

**Figure 9:**
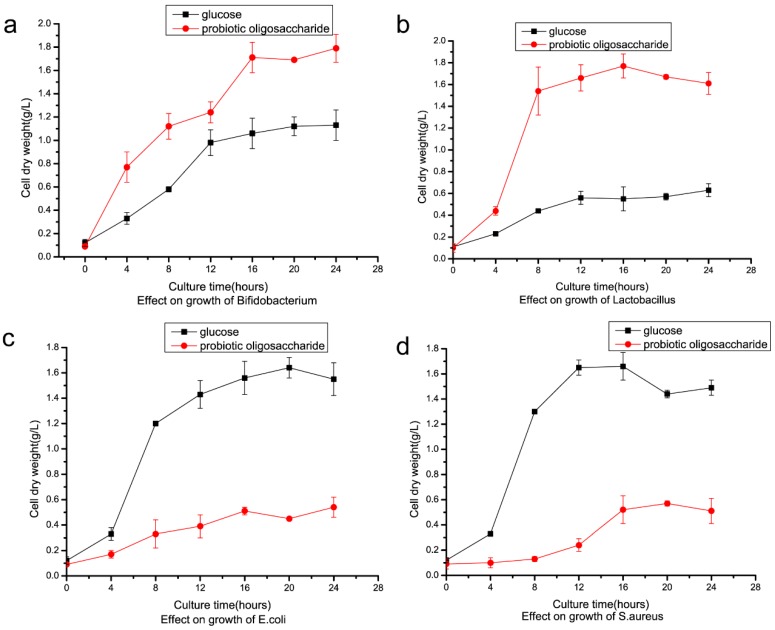
Effect of IMO on growth of microorganisms. (**a**) Effect on growth of *Bifidobacterium*; (**b**) effect on growth of Lactobacillus; (**c**) effect on growth of *E.coli*; (**d**) effect on growth of *S.aureus*. Probiotic oligosaccharide means IMO.

**Figure 10:**
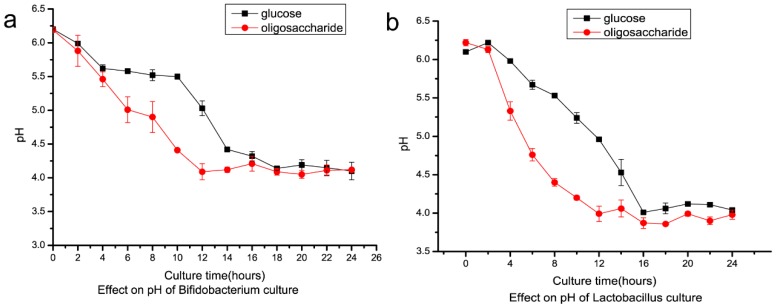
Effect of IMO on the pH of the microorganism culture. (**a**) Effect on pH of *Bifidobacterium* culture; (**b**), effect on pH of *Lactobacillus* culture

IMO is a prebiotic, which exerts positive effects on human intestinal flora. IMO prepared in this study was used as a carbon source in four microorganism strain culture tests. *Bifidobacterium longum* and *Lactobacillus rhamnosus* growth improved when using IMO as a replacement for glucose as the carbon source. After 28 h of culturing the cell, the dry weight of *Bifidobacterium longum* reached 1.74 g/L and 1.06 g/L using IMO and glucose, respectively (Figure 9a). For *Lactobacillus rhamnosus,* cell dry weight improved to 1.73 g/L from 0.52 g/L after 16 h of culture when IMO replaced glucose (Figure 9b). On the other hand, *E. coli* and *S. aureus* grew worse when using IMO to replace glucose as the carbon source (Figure 9c,d). Cell dry weight of *E. coli* reduced to 0.38 g/L from 1.58 g/L after 16 h of culture using IMO to replace glucose. Cell dry weight of *S. aureus* reduced to 0.50 g/L from 1.62 g/L in the same conditions. *Lactobacillus rhamnosus* and *Bifidobacterium longum* are considered intestinal probiotic microorganisms, and *E. coli* and *S. aureus* are common foodborne pathogenic bacteria that cause gastrointestinal diseases. Cell dry weight of *Lactobacillus* and *Bifidobacterium* increased when they were cultured using IMO instead of glucose as the carbon source (Figure 9a,b). Because only *Lactobacillus* and *Bifidobacterium* could utilize these nondigestible oligosaccharides with α-1,6 and α-1,3 glycosidic bonds, *E. coli* and *S. aureus* could not utilize oligosaccharides, due to the lack of the related hydrolytic enzyme, and their growth was inhibited (Figure 9c,d). When *Lactobacillus* and *Bifidobacterium* utilized the isomalto-oligosaccharide, they could synthesize many short-chain fatty acids (SCFAs), which are beneficial to the host organism. This is one of the mechanisms of this kind of prebiotic. The pH of culture of IMO was more acidic compared to the culture when glucose was the carbon source (Figure 10a,b). After 12 h of culture, *Bifidobacterium* reached pH 4.7 and 5.6 using IMO and glucose, respectively. For *Lactobacillus,* the pH of the culture using IMO and glucose reached 4.1 and 5.0 after 12 h, respectively. The acidic condition was beneficial for the growth of the two bacteria.

## 3. Discussion and Conclusions

DexKQ dextranase from *Arthrobacter oxydans* KQ11 is a new member of the GH49 family of enzymes. It is an alkaline dextranase with a broad pH adaptation range (pH 5.0–13.0) and high catalytic activity (*k*cat/*K*m of 3.03 s^−1^ μM^−1^). Most known GH49 dextranases are acidic and derived from fungus. A few GH49 dextranases have been found from terrestrial microbes. DexKQ was the first recombinantly expressed GH49 dextranase, whose coding gene was from marine bacteria. Its high catalytic activity in a wide pH range and its stability in alkali environments make it a promising dextranase for novel industrial applications in alkaline conditions. Because of the special composition of DexKQ products, its molecular catalytic mechanism is worth studying. In our in vitro study, isomalto-oligosaccharide (IMO) prepared using DexKQ recombinant dextranase could improve the growth prebiotic bacteria of *Lactobacillus* and *Bifidobacterium* and inhibit the pathogenic bacteria *E. coli* and *S. aureus.* In a new report, co-administration of green tea extract with IMOs could prevent high-fat diet-induced metabolic alterations via preventing gut dysbacteriosis in mice. SCFAs as metabolites of IMOs are produced by intestinal bacteria and play important roles in flora regulation [34]. At present, IMO is commercially manufactured by immobilization of α-glucosidase using starch as the substrate [35,36]. IMO sold on the market had the main components of isomaltose, panose, and a small amount of isomaltriose, and it was produced by this technical route (Figure 8). Isomaltose oligosaccharides of various polymerization degrees would have more potential functions, but it has been difficult to produce other polymerization degrees of oligosaccharides using this route. Enzymatic transformation of dextran into IMO using DexKQ dextranase offers a new technical route for production of IMOs with higher polymerization degrees. It would be convenient to produce IMOs of specific molecular weights by controlling the enzyme dose and catalytic conditions. IMO is a promising prebiotic that is receiving more attention. When intestinal *Lactobacillus* and *Bifidobacterium* utilized isomalto-oligosaccharides, they could synthesize many short-chain fatty acids (SCFAs), which are beneficial to the host organism. Some animal experimental results confirmed SCFA could modulate gene expression of intestinal epithelial cells and repair the epithelial barrier [37]. Some reports have indicated that short-chain fatty acids (SCFAs) could modulate the composition and activity of intestinal microbiota [38]. All the above findings constitute the mechanisms of this oligosaccharide prebiotic. In our study, because SCFAs were produced, the pH of the IMO culture was more acidic compared to the culture when glucose was the carbon source (Figure 10a,b). After 12 h of culture, *Bifidobacterium* reached pH 4.7 and 5.6 using IMO and glucose, respectively. For *Lactobacillus,* the pH of the culture using IMO and glucose reached 4.1 and 5.0, respectively, after 12 h. Other nondigestible oligosaccharides (NDOs) like fructo-oligosaccharides and xylo-oligosaccharides also could be utilized by intestinal *Lactobacillus* and *Bifidobacterium* to produce SCFAs. Other NDOs usually have a laxative effect when taken at a high dosage. However, IMOs are tolerated at higher doses compared to other NDOs [39]. IMOs with various polymerization degrees could have more applications in health food in the future.

## 4. Materials and Methods

### 4.1. Molecular Cloning

Purified native dextranase protein from *Arthrobacter oxydans* KQ 11 culture was prepared in a previous experiment. MALDI-TOF-MS analysis of protein bands was conducted by BoYuan Biotech Company (Shanghai, China). Amino acid sequence analysis was conducted online by Mascot software (www.matrixscience.com). Whole-genome sequencing of *Arthrobacter oxydans* KQ 11 and data analyses took place at Novogene (Beijing, China). The gene-coding target dextranase in *Arthrobacter oxydans* KQ 11 was confirmed by analyzing the results of MALDI-TOF mass and genomic sequencing.

### 4.2. Bioinformatic Analysis of the Dextranase Gene and Protein Sequence

MEGA 5.0 and the neighbor-joining method were used to construct the phylogenetic tree. Nucleotide sequences of the new dextranase gene were analyzed by DNASTAR software (www.dnastar.com). A signal peptide of this dextranase was predicted by the Signal IP 4.1 server (www.cbs.dtu.dk). Its amino acid sequence was aligned with other dextranase sequences using Clustal X 1.83. Conserved amino acids of dextranase were found in the alignment results of the DexKQ sequence and marked with black triangles. The crystal structure was download from the website PDB (www.rcsb.org) and analyzed by PyMOL software (www.pymol.org). Secondary structure predictions based on three-dimensional modeling were accomplished using the ESPript3.0 network station.

### 4.3. Recombinant Expression of Dextranase

Recombinant expression and protein purification protocols were carried out according to the Molecular Cloning Handbook [40]. The pET28a vector and BL21(DE3) host cell were used in recombinant protein expression. The sequences of primers used in construction of the expression vector were KQ-28aF: GGGAATTCCATATGAAGCATTACCTCCGTCTA; KQ-28aR: CCCAAGCTTCC-ACGCGTTCCAGTTATCCCA. In the protein expression procedure, 1 mM working concentration of isopropyl β-D-Thiogalactoside (IPTG) was added into the recombinant *E. coli* culture as the inducer. After 12 h of induction at 28 °C, recombinant DexKQ dextranase with c-terminal 6× his tag was purified by Ni-NTA resin.

### 4.4. Dextranase Activity Assay

Dextranase activity was measured by the DNS (3,5-dinitrosalicylic acid) method. Purified enzyme preparation was diluted 100 times using dd H_2_O. The substrate was 3% (w/v) dextran T70 with a molecular weight of 70,000 Da dissolved in 20 mM phosphate salt buffer (pH 8.0). Diluted enzyme samples of 100 μL were mixed with 100 μL of substrate solution. After incubating at 50 °C for 30 min, 200 μL of DNS reagent was added into the mixture to stop the reaction. After boiling at 100 °C for 10 min, 3.0 mL of ddH_2_O was added into the mixture. The reducing sugar concentration was calculated by the absorbance value of the mixture at 540 mm. One unit (1 U) of dextranase activity was defined as the quantity of enzyme that could hydrolyze dextran into reducing sugar, equivalent to 1 μmol isomaltose/min, at the above assay conditions. The isomaltose standard curve was constructed using the above testing method. Protein concentration (mg/mL) was measured by the Bradford method. Commercial dextranase (Sunshine Co. Ltd., China) was used for comparisons. In its specific activity test reactions, conditions were the same as the standard protocols, except the buffer was changed to 50 mM HAc-NaAc (pH 5.0), which was the recommended reaction buffer.

### 4.5. Effect of pH on Dextranase Activity and Stability

Dextranase activity at different pH conditions was determined at a pH range of 3.0 to 13.0 (HAc-NaAc buffer, pH 3.0–6.0; phosphate buffer, pH 6.0–8.0; glycine-NaOH buffer, pH 8.0–13.0). pH stability of dextranase was tested by incubating the enzyme in the above buffers in a pH range of 3.0 to 14.0 at 25 °C for 2 h. NaOH solution (0.1 M) was used as a pH 14.0 buffer. The residual enzyme activity was determined at the above standard assay conditions.

### 4.6. Effect of Temperature on Dextranase Activity and Stability

Effects of temperature on dextranase activity were evaluated at a range of 30–70 °C. Thermal stability of dextranase was evaluated by incubating the enzyme in 50 mM phosphate salt buffer (pH 8.0) for 2 h at different temperatures. The commercial dextranase buffer was changed into 50 mM HAc-NaAc buffer (pH 5.0). The residual enzyme activity was determined at standard assay conditions.

### 4.7. End Products Analysis

The end product test was operated at 50 °C for 30 min in 50 mM phosphate salt buffer (pH 8.0) with 3% dextran T70 (*w*/*v*). The hydrolysis end products were analyzed by thin-layer chromatography (TLC) using a silica gel 60 plate. The solvent system consisted of 1-butanol/pyridine/water (6:4:3, *v*/*v*/*v*). Glucose and isomaltose were used as the standards. After chromatography, the plate was sprayed with 10% (*v*/*v*) sulfuric acid in ethanol and heated at 130 °C for 5 min to visualize oligosaccharide spots.

### 4.8. Enzyme Kinetics Assay

Dextran T70 with different concentrations of 0.1–2% (*w*/*v*) were used as standard substrates. The DexKQ reaction buffer was a 50 mM phosphate salt buffer (pH 8.0). The commercial dextranase reaction buffer was changed into 50 mM HAc-NaAc (pH 5.0). Enzyme activity assay proceeded at the same conditions as in the above dextranase activity assay. Michaelis constant, Km, and catalytic constant, kcat, values were calculated from Lineweaver–Burk plots.

### 4.9. Preparation of Isomalto-Oligosaccharide (IMO)

In preparation of isomalto-oligosaccharide, 100 g dextran T20 dissolved in 1 L of phosphate salt buffer (50 mM, pH 8.0) was used as the substrate. Recombinant DexKQ enzyme of 300 U was added into the reaction mixture. After incubation at 50 °C for 12 h, hydrolysis was stopped by incubation at 100 °C for 10 min. Next, the reaction mixture was concentrated and desalinated using small nanofiltration equipment (Hangzhou Donan Memtec Co., Ltd. Hangzhou, China). Lastly, the condensed sugar solution was lyophilized into powder.

### 4.10. Application of IMO in Culturing Microorganisms

Four microorganism strains were used in this test. *Lactobacillus rhamnosus* ATCC 53103 was grown in Man Rogosa Sharpe (MRS) medium using static liquid culture. *Bifidobacterium longum* ATCC 15707 was grown in MRS medium using anaerobic liquid culture. *Escherichia coli* ATCC 25922 and *Staphylococcus aureus* ATCC 6538 were cultured in Luria-Bertani (LB) medium while shaking the liquid culture with a rotating speed of 180 rpm. Glucose and IMO were filtration-sterilized and added into medium separately at a working concentration of 5 g/L. For the four strains, the other culture conditions were the same. Medium (50 mL) was put into 250 mL flask, and the culture temperature was set to 37 °C. During culture, bacterial cell samples were collected by centrifugation at 4 h intervals and dried to a constant weight at 60 °C in a vacuum drying oven. The pH of the bacterial culture was tested at 2–4 h intervals.

## Figures and Tables

**Table 1:** Internal peptide sequence of native dextranase and amino acid (AA) sequence of ORF4194.

Internal Peptide Sequences of Native Dextranase Enzyme	Partial AA Sequence of ORF 4194
1: AYDAFTYMSIPR	SFYDLQVAQENQPDKAYDAFTYMSIPRSGKDKIWVDVSLRTGQTITSADQVQIRPSSYNFEKQLVDADTVKIKVPYSDAGYR FSVEFEPQLYTAYNDMSGDSGKLTTEAEGNRAIHTEPRNSMMIFAEPKLRGEQKERLVPTEESGSIHQVGSWYWQTDGIELYKGST
2: TGQTITSADQVQIRPSSYNFEK	
3: IKVPYSDAGYR	
4: FSVEFEPQLYTAYNDMSGDSGK	
5: NSMMIFAEPK	
6: QVGSWYWQTDGIELYK	

**Table 2:** Comparison of conserved key amino acid sites in dextranase DexKQ and dextranase DEX49A.

DEX49A	DexKQ	DEX49A	DexKQ
Asp395	Asp439	Tyr463	Trp507
Asp376	Asp420	Trp267	Trp304
Asp396	Asp440	Tyr269	Tyr306
Asn481	Asn529	Trp568	Trp630
Asn507	Asn555	Trp571	Trp633
Asn417	Asn461	Thr375	Thr419
Trp425	Trp469	Gly377	Gly421

**Table 3:** Summary of catalytic properties of some reported dextranases.

Origin	Temperature and pH Parameters	Classification	Main End Product	Reference
*Arthrobacter globiformis* T-3044 *E. coli* recombinant	unknown	Endodextranase GH49	unknown	[6]
*Arthrobacter globiformis* CB-8 *E. coli* recombinant	unknown	Endodextranase GH49	unknown	[7]
*Arthrobacter oxydans* *E. coli* recombinant	optimum activity at 37 °C, pH 7.5	Endodextranase GH49	IMO2,3	[8]
*Arthrobacter oxydans* *E. coli* recombinant	stable below 40 °C and optimum activity at pH 6.0	Endodextranase GH49	unknown	[9]
*Thermotoga lettingae* TMO	optimal pH and temperature were 4.3 and 55–60 °C stable from pH 4.3–10.0	Endodextranase GH66	IMO2,3	[15]
*Paenibacillus* sp. *E. coli* recombinant	optimal pH and temperature were 5.5 °C and 60 °C	Endodextranase GH66	IMO2,3	[16]
*Paenibacillus* sp. native	optimal pH was 5.5	Endodextranase GH66	IMO4	[17]
*Streptococcus mutans* *E. coli* recombinant	optimal pH and temperature were 5.0 °C and 40 °C	Endodextranase GH66	IMO2,3,4	[18]
*Thermoanaerobacter pseudethanolicus* *E. coli* recombinant	optimal pH was 5.2 and a half-life of 7.4 h at 70 °C between pH 3.1 and 8.5	Endodextranase GH66	IMO2,3	[19]
*Arthrobacter globiformis* T6 *E. coli* recombinant	optimal pH and temperature were 3.5 °C and 60 °C	Isomaltose exo-dextranase GH27	IMO2	[20]
*Arthrobacter globiformis* I42 *E. coli* recombinant	optimum pH and temperature were 6.0 and 45 °C	Glucodextranase GH15	unknown	[21]
*Bacillus circulans* T-3040 *E. coli* recombinant	optimal pH and temperature were 5.2 °C and 60 °C	Cyclodextran glucanotransferase GH66	cyclodextran	[22]
*Talaromyces pinophilus* native	optimum temperature of 45 °C and an optimum pH of 6.0, stable over pH range 3.0 to 10.0	Endodextranase GH49	IMO2,3	[23]
*Chaetomium erraticum* native	optimal pH and temperature were 5.2 °C and 60 °C	Endodextranase GH49	IMO2,3	[24]
*Penicillium minioluteum* *Pichia pastoris* recombinant	optimum activity at pH 5.0 and 60 °C	Endodextranase GH49	IMO2,3	[25,26]
*Penicillium funiculosum* *recombiant in other Pencillium strain*	activity optimal at pH 4.5–5.0 and 55–60 °C	Endodextranase GH49	Glucose and IMO2	[27]
*Lipomyces starkeyi* *S. cerevisiae* recombinant	maximum activity at pH 6.0 and 37 °C	Endodextranase GH49	IMO2,3	[28]
*Fusarium* sp. native	maximum activity at pH 6.5 and 35 °C, stable under 4 °C and at pH ranging from 4.5 to 11.8	Endodextranase	IMO3	[29]
*Catenovulum* sp. native	maximum activity at pH 8.0 and 40 °C, stable under 30 °C and pH ranging from 5.0 to 11.0	Endodextranase GH49	IMO2	[30]
*Streptomyces* sp. *NK458* native	maximum activity at pH 9.0 and 60 °C stable at pH 5.0–10.0	unknown	unknown	[2]
*Streptomyces anulatus* native	retained 50% of initial activity at pH 5.1–10.1 under 30 °C for 1.5 h	Endodextranase GH49	unknown	[31]
*Arthrobacter oxydans* KQ11 *E. coli* recombinant	maximum activity at pH 9.0 and 55 °C, stable under 40 °C and at pH ranging from 6.0 to 13.0	Endodextranase GH49	IMO4,5,6	This study

**Table 4:** Substrate specificity of DexKQ and commercial dextranase.

Substrate	Main Linkage	Relative Activity (%)	
		DexKQ Dextranase	Sunshine Dextranase
3%Dextran T5	α-1,6	70.5 ± 2.1	102.5 ± 0.8
3% Dextran T11	α-1,6	44.4 ± 0.3	126.6 ± 1.5
3% Dextran T20	α-1,6	90.5 ± 0.5	101.2 ± 1.3
3% Dextran T40	α-1,6	85.0 ± 2.3	152.5 ± 0.4
1% Dextran T70	α-1,6	55.0 ± 1.2	33.1 ± 0.5
3% Dextran T70	α-1,6	100.0	100.0
5% Dextran T70	α-1,6	76.3 ± 0.4	116.4 ± 1.2
8% Dextran T70	α-1,6	87.4 ± 0.5	94.5 ± 1.6
10% Dextran T70	α-1,6	181.2 ± 1.4	303.0 ± 0.4
15% Dextran T70	α-1,6	120.8 ± 0.4	163.5±1.5
20% Dextran T70	α-1,6	147.8 ± 1.1	485.9 ± 2.3
3% Dextran T100	α-1,6	71.9 ± 4.5	78.734 ± 3.1
3% Dextran T200	α-1,6	76.3 ± 3.3	90.5 ± 0.6
3% Dextran T500	α-1,6	85.9 ± 0.3	75.2 ± 2.2
Blue dextran 2000	α-1,6	75.6 ± 1.1	67.3 ± 0.9
pullulan	α-1,6	0	0
laminaran	β-1,3	0	0
Amylose	α-1,4	0	0
Amyloid	α-1,4 and α-1,6	0	0
Sucrose	α-1,4	0	0
Trehalose	α-1,2	0	0
Carboxymethyl cellulose	β-1,4	0	0

The activity was measured using various concentrations of substrate in 50 mM NaH_2_PO_4_/Na_2_HPO_4_ buffer (pH 6.0) at 50 °C for 30 min; The results reported are the means of three replications ± SD.
